# Period poverty and mental health in a representative sample of young women in Barcelona, Spain

**DOI:** 10.1186/s12905-023-02328-w

**Published:** 2023-04-28

**Authors:** Marga Marí-Klose, Albert Julià, Sandra Escapa, Pedro Gallo

**Affiliations:** Department of Sociology, University of Barcelona, Barcelona, Spain

**Keywords:** Mental health, Period poverty, Youth, Quantitative research methodology, Representative sample

## Abstract

**Background:**

The intersection between poverty and mental health is clear. Period poverty, understood as the lack of access to menstrual products, has been gaining attention especially among low and middle-income countries as an overlooked aspect of gendered poverty. Less is known about the incidence of period poverty in high-income countries and its association with mental health. The purpose of this study is to examine this association in a representative sample of young women living in an urban setting in southern Europe.

**Methods:**

This is a cross-sectional study. Data were obtained from a representative survey of individuals aged 15 to 34 in the city of Barcelona (Spain), with a sample group of 647 young women. Subjects were selected through a systematic stratified random sampling method. A proportional quota sampling was used. The information was registered using CAPI data collection method. Period poverty was measured by a combination of three questions about the lack of access or misuse of menstrual products for economic reasons. The GHQ-12 was used to measure the risk of poor mental health. The analysis was carried out using multivariable logistic regression.

**Results:**

From our sample, 15.3% of young women reported having experienced period poverty. Higher odds of poor mental health were estimated for women facing period poverty (AOR = 1.85 p < 0.05). This effect is statistically significant after controlling by their income status and level of deprivation. Young women living in poorer households have a higher probability of poor mental health than those living in high-income households (AOR = 0.47 p < 0.05). Finally, material deprivation was associated to an increased risk of poor mental health among young women reporting period poverty (AOR = 2.59 p < 0.01).

**Conclusion:**

We found that a considerable number of young women living in an urban setting in a high-income country cannot afford menstrual products, and this may have an impact on their mental wellbeing. The relationship between period poverty and respondents’ mental health is significant when controlling for factors known to confer an increased risk of poor mental health. If confirmed by further research, the public health burden of poor mental health in young women could be reduced by policy-level interventions to improve access to menstrual products.

## Background

One of the most consistently replicated findings in social sciences has been the association between mental health problems and poverty or socio-economic disadvantage [1, 2]. Poverty is related to volatile income and expenditure patterns. The resulting worries and uncertainty to meet one’s basic needs may worsen mental health. In this respect, several categories have emerged recently to describe the multidimensional nature of poverty and its direct or indirect impact on mental health, including food poverty [3, 4], housing poverty [5], and energy poverty [6, 7]. This article explores an overlooked aspect of gendered poverty, frequently labelled as “period poverty” [8], that is, not being able to afford menstrual products and its association with mental health outcomes. “Period poverty” here may be understood as an additional category that intersects with other dimensions of poverty, but adding a distinctive gendered and body nature, we aim at disclosing in this paper.

Only recently, menstruation, and menstrual health and management in particular, have become a pressing issue in the public health debate, increasing investment to overcome the menstruation related barriers in schools in low and middle-income countries, and more recently, spreading the “menstrual equity” and “period poverty” movements across high-income countries [9, 10]. However, to the date, there are few studies identifying and measuring the outcomes of period poverty in high-income countries. Particularly in low-income countries, inadequate menstrual hygiene has been associated to poor health-related quality-of-life and adverse reproductive health outcomes such as infertility, recurrent abortions, ectopic pregnancies, and bacterial infections [11]. In addition, the lack of access to appropriate protection may cause women to feel discomfort, embarrassment and shame [10]. Such feelings and perceptions may lead to self-exclusion from daily life activities such as education or work, which surely have significant implications for women’s equal opportunities [12].

While the majority of research on menstrual hygiene needs focuses on women living in low and middle-income countries, recent research suggests period poverty is also experienced by women in high-income countries [13–19]. However, most of the studies and grey literature published with reference to high-income countries are qualitative studies, largely using interviews and focus group techniques to uncover perceptions, views and attitudes towards period poverty [13, 17, 18, 20, 21]. A study published recently, using a representative sample of the young population living in the city of Barcelona, conveyed that 15.3% of 16–34 years-old women reported facing financial or other barriers to an adequate management of their menstruation. The percentage was especially high among those who were economically dependent and lived with their parents or relatives (21.4%) [22]. Other, rather few, published studies based on non-representative samples in high-income settings report similar findings [19, 23–25]. Further, as a result of the coronavirus pandemic and subsequent lockdown, several NGOs and charity organisations in high-income countries have reported increasing numbers of women struggling financially to access menstrual products [25–27]. Despite a number of relevant policy decisions in Canada, US, UK (England and Scotland), Australia and Spain (Catalonia) have been put forward to facilitate access to such products, there is a need of relevant survey data using representative samples of the population that could help to better understand the complex nature of the problem and enrich the debate on the issue.

Analyzing the potential association between period poverty and mental health is particularly important in the youth population. Youth is the stage of life in which most mental disorders begin [28]. In addition, young people experience a high rate of self-harm, and suicide is reported as a leading cause of death among the young population. There is a strong relationship between poor mental health and many other health and development concerns among young people, notably with educational achievements, substance use and abuse, violence and reproductive and sexual health [29]. Furthermore, the COVID-19 pandemic has exposed youth to a higher risk of negative psychological and social effects [29–31]. A recent survey on the effects of COVID-19 on mental health in Spain shows the negative effects of the pandemic by age groups. Young people from 18 to 24 years old and from 25 to 34 are the two age groups with the worse mental health indicators [32]. Young age also represents a stage in life where the confluence of social risks may be high as young individuals go through important life episodes that will determine their present and future life [33]. Further, various international reports have found poverty and social exclusion rates are concentrated in the early stages of the life course [34]. In this regard, period poverty may well be situated within the wider problem of the growing risk of poverty particularly among children and young adults in Spain as well as in other high-income countries [35, 36].

To date, despite the link between unmet needs and mental health, and the importance of shame and stigma associated to menstruation, very little research examines how unmet menstrual needs in high-income countries are associated to mental health. To our knowledge, two scientific publications have addressed this issue, one in the US and the other in France. Cardoso et al. [15] reported an association between period poverty and moderate/severe depression among a nationally drawn sample recruited online (N = 471) of young college-aged women in the U.S. The authors point out some of the limitations of the study such as the difficulty to measure income levels among college students (not included as a covariate). As a result, they were not able to test whether period poverty was associated with depression regardless of economic deprivation. The study sample was not probabilistic-based, and hence, not representative of the population or a specific group of that population, which means their findings are not generalizable. In France, Gouvernet et al. [16] also observe an association between period poverty and mental health. In this case, they investigated the relationship between period poverty and anxiety/depression between women aged 18 to 50 in the context of the first COVID-19 lockdown with a non-representative sample also recruited online (N = 890). The authors detected an overrepresentation of a privileged background and young adults among participants.

In the light of previous published research, the objective of our paper is twofold: (1) to provide the first published data examining the association between period poverty and mental health using a representative sample of young women in an urban context in southern Europe during the pandemic, and (2) to overcome some of the limitations of previous published research including income, labour status and level of deprivation as covariates in the analysis. To our knowledge, this is the first quantitative analysis to be published on the association between period poverty and mental health using a representative sample of the population. Filling the current knowledge gap around this issue is crucial to the design and implementation of more effective policies fighting poverty and gender discrimination.

## Methods

This is a cross-sectional study. To carry out this research, we used the database of the *2020 Youth Survey* in Barcelona (*Enquesta a la Joventut de Barcelona*), a representative survey of individuals aged 15 to 34 in the city of Barcelona. The survey sample was comprised of 1,407 subjects who were selected through a stratified systematic random sampling method from the municipal census. Data were collected in the period March-June 2020, avoiding the lockdown period. Interviewers visited a number of households randomly selected according to a set of route maps in different neighbourhoods in Barcelona. The households were previously identified for each interviewer, and the selected individuals were informed on the purpose of the survey and all agreed to participate. The sample was first stratified by the household average income of the neighbourhood of residence (low, middle, and high income). Then crossed quotas were established by nationality (Spanish and others), by sex and age (15 to 19 years, 20 to 24 years, 25 to 29 years, and 30 to 34 years) following the distribution of the study population. Proportional quota sampling was used. The margin of sampling error is ± 2.6% for a 95% confidence level and p = q = 0.5. The sample group of young women in this study represented 50.5% (n = 711) of the initial sample. The selection of variables used in the regression models lowered the sample to 647 women aged 15 to 34. A descriptive analysis was performed to verify that the lost cases (n = 64) do not correspond to any specific social profile.

The information was registered using CAPI data collection method (Computer-Assisted Personal Interviewer). The interviewer wrote down the answer in most of the questionnaires unless questions were sensitive or intimate (e.g. period poverty questions or alcohol/tobacco/cannabis consumption questions). The interviewee answered directly these particular questions without the supervision of the interviewer.

Using logistic regression analysis, we disentangle which is the effect of experiencing period poverty on the risk of poor mental health among young women. All data analyses were processed with SPSS25. Our dependent variable is the *Risk of poor mental health* based on the GHQ-12 scale (General Health Questionnaire). The GHQ-12 is an easy, short, reliable valid instrument for assessing mental health status [37] consists of 12 questions on aspects related to mental health and psychological distress. The answer categories from the GHQ-12 scale were scored according to the method proposed by its author, with the first two response options having a score of 0 and the last two having a score of 1 (0-0-1-1). A cut-off ≥ 3 points was selected to indicate a risk of poor mental health in the population following the author’s recommendation [38].

The *period poverty* variable was created from a set of three questions based on the Mayor of London-YouGov pioneer research from the London City Council [24]. These questions are: “Have you ever had difficulties in accessing products for your menstrual hygiene (pads, tampons, menstrual cup, etc.) for economic reasons? Have you ever used some menstrual hygiene products longer than recommended because you had no replacement? Have you ever used other products, not specific for menstruation (WC paper, towels, etc.), for economic reasons?”. All three questions have dichotomous answers (1 = Yes, 0 = No). Thus, the value “1” in the period poverty indicator include those who had experienced any of these three situations, and “0” if they experienced none of them.

Our main socioeconomic variable is *Household equivalent income*. This variable is calculated by dividing the household income by the household size, according to OECD’s adjusted consumption unit [39]. We entered the variable into the model in quartiles (Q1 is the lower income quartile and is the reference category). We decided to add a fifth category of “No information” due to the high percentage of missing data in the household income variable (30.4% of subjects do not report household income). Some studies show the association between having lower income and higher risk of psychological distress, especially in a COVID-19 pandemic context [40–42]. According to these studies, our main hypothesis is that we expect higher risks of poor mental health among poorer young women, and specifically among those experiencing period poverty.

We also introduced in the analysis the *material deprivation scale* variable as a non-monetary indicator of life conditions based on the EU-SILC set of material deprivation indicators [43]. Material deprivation is another factor associated with poorer mental health [44], even after controlling for income poverty [45]. The material deprivation scale is measured by five items: (1) “Do you consider that your household has the capacity to cope with an unexpected expense of 700 euros with its own resources?”; (2) “Can the household afford to go on holiday away from home for at least one week a year?”; (3) “Can you afford to eat meat, chicken or fish (or equivalent for vegetarians), at least every two days?”; (4) “Are you able to keep your home adequately warm?”; (5) “Have you had any delay in the payment of the rent or mortgage, or in the payment of any of the household supplies (water, gas, electricity) in the last 12 months?”. The final material deprivation scale indicator is a sum of these five items of deprivations and is recoded in five categories (“0” identifying no material deprivation and used as a reference category, “1 item”, “2 items”, “3 or more items”, and “No information”).

In our models we also include variables in order to capture the level of individual economic autonomy. The *labour status* variable has six categories according to women’s main labour market situation at the time of the survey (“studying” as a reference category, “working”, “unemployed”, “ERTE” –this is the Spanish acronym of Record of Temporary Employment Regulation, that is, temporal layoff–, “Inactive”, and “No information”). Recent evidence based on a systematic review of the literature showed an association between unemployment among young people and mental health, although the causal relationship is less clear [46]. So, according to previous evidence, it is plausible that unemployment may impact women’s mental health [47, 48].

“*Living arrangement”* was measured using information about who the young women live with. Respondents were categorized as “alone” as a reference category, with her “father and/or mother” and other family members or non-family members, only with her “partner”, “partner and children”, with “friends and or flatmates” -with or without partner-, “other family members”, and “other no family members”. According to Kwong et al. [49], living alone during the pandemic was also associated with higher depression in different cohorts, but was not associated with anxiety.

We also added a set of controls variables measuring socio-demographic characteristics: *Age* (with four categories: “15–19 years old”, “20–24”, “25–29”, and “30–34”), *Country of origin* (“Spain” as a reference category, “other EU country”, “Latin America”, and “Other”), or S*elf-reported health status* (“excellent/very good/good” as a positive health status, and “fair/poor” as a negative health status and reference category) [15, 50, 51].

## Results

Table 1 provides the main characteristics of the study sample. Up to 45.1% of 15–34 years-old women in Barcelona reported having poor mental health according to the GHQ-12 **> = 3** indicator. And 15.3% of young women reported facing financial or other barriers to adequate management of their menstruation. Further, the prevalence of risk of poor mental health is higher among women with period poverty (62.6%) than those without this kind of deprivation (50.0%). Major differences are found in respect to labour status (67.7% of unemployed women are at risk of poor mental health), income level (63.1% of those in the first quartile of equivalent income are at risk of poor mental health), number of household material deprivations (two items 50.6%, three or more 70.9%), and self-reported health (fair/poor self-reported health 82.6% at risk of poor mental health).

**Table 1:** Characteristics of young women (15–34 y. o.) and the association with the risk of poor mental health (GHQ-12 > = 3) (N = 647)

	Sample			Poor mental health			Sample			Poor mental health	
	%	N		%	N		%	N		%	N
**GHQ-12 ≥ 3**						**Labour status**					
Yes	45.1	292				Studying	32.6	211		39.8	84
No	54.9	355				Working	48.7	315		39.0	123
**Period poverty**						Unemployed	10.1	65		67.7	44
Yes	15.3	99		62.6	62	Inactive	3.5	23		52.2	12
No	84.7	548		50.0	230	ERTE	4.0	26		57.7	15
**Self-rated health status**						No info	1.1	7		71.4	5
Positive	91.0	589		41.4	244	**Income**					
Negative	9.0	58		82.6	48	Q1	17.1	111		63.1	70
**Age**						Q2	16.7	108		45.3	49
15 to 19 years	16.7	108		42.6	46	Q3	18.2	118		39.8	47
20 to 24 years	21.5	139		43.9	61	Q4	17.2	111		31.5	35
25 to 29 years	30.9	200		44.5	89	No info	30.8	199		41.3	82
30 to 34 years	30.9	200		48.0	96	**Material deprivation scale**					
**Origin**						None	43.7	283		37.1	105
Spain	55.5	359		43.2	155	One item	21.8	141		36.9	52
Other EU countries	7.4	48		56.3	27	Two items	12.8	83		50.6	42
Latin America	31.4	203		45.3	92	Three or more items	13.3	86		70.9	61
Other	5.7	37		48.6	18	No info	8.4	54		42.6	23
**Living arrangements**											
Alone	9.1	59		49.2	29						
Father/mother	33.4	216		46.3	100						
Partner	21.6	140		37.9	53						
Partner and children	7.6	49		42.9	21						
Friends/flatmates	17.6	114		45.6	52						
Other family members	9.3	60		56.7	34						
Other no family members	1.4	9		33.3	3						

The logistic regression analysis shown in Table 2 confirms our main hypothesis. Young women who reported experiencing period poverty were significantly more likely of being at risk of poor mental health (AOR = 1.85 p < 0.05). This effect is statistically significant after adjusting the model by income status and the number of deprived items in the household. Young women living in the poorest household (Q1) are exposed to a higher probability of poor mental health risk relative to those living in high-income households (Q4 AOR = 0.47 p < 0.05). There are also statistically significant differences between those women living in households with no material deprivation and those lacking three or more items (AOR = 2.59 p < 0.01).

**Table 2:** Logistic regression predicting prevalence of risk of poor mental health (GHQ-12 > = 3) for young women (15–34 y.o.) (N = 647)

	AOR	(95% C.I.)
**Period poverty** (Ref. No)		
Yes	1.85*	(1.12–3.05)
**Self-rated health status** (Ref. Negative)		
Positive	0.21***	(0.98 − 0.43)
**Age** (Ref. 15 to 19 years)		
20 to 24 years	1.08	(0.59–1.97)
25 to 29 years	1.28	(0.62–2.66)
30 to 34 years	1.48	(0.69–3.19)
**Origin** (Ref. Spain)		
Other EU countries	1.64	(0.85–3.16)
Latin America	0.81	(0.53–1.23)
Other	1.09	(0.51–2.31)
**Living arrangements** (Ref. Alone)		
Father/mother	1.25	(0.61–2.58)
Partner	0.82	(0.41–1.64)
Partner and children	0.63	(0.25–1.63)
Friends/flatmates (with or without partner)	1.07	(0.53–2.18)
Other family members	1.09	(0.47–2.50)
Other no family members	0.45	(0.09–2.13)
**Labour status** (Ref. Studying)		
Working	1.18	(0.70–1.99)
Unemployed	2.46*	(1.13–5.33)
Inactive	0.99	(0.29–3.35)
ERTE	1.70	(0.60–4.83)
No info	3.07	(0.47–20.17)
**Equivalent income** (Ref. Q1)		
Q2	0.54	(0.28–1.02)
Q3	0.64	(0.33–1.23)
Q4	0.47*	(0.23–0.98)
No info	0.59†	(0.34–1.03)
**Material deprivation scale** (Ref. None)		
One item	0.92	(0.58–1.46)
Two items	1.57	(0.90–2.73)
Three or more items	2.59**	(1.33–5.06)
No info	1.06	(0.55–2.05)
**Constant**	2.98†	(0.92–9.61)
**Pseudo R** ^**2**^	0.11	
**Log pseudolikelihood**	-394.99	

AOR: adjusted odds ratio; CI: Confidence Interval; Ref.: Reference category.

Significance levels: †p < 0.1, *p < 0.05, **p < 0.01, ***p < 0.001.

No differences were found between living alone and other living arrangements, origin, and the age of young women. However, being unemployed rather than studying is significantly associated with being at risk of poor mental health (AOR = 2.46 p < 0.05). In other models not included here, being unemployed has a higher probability of risk of poor mental health than being at work (AOR = 2.09 p < 0.05) when “working” is tested as a reference category, however we did not find significant differences whit other labour status categories. We also found that self-reported health status is significantly associated to mental health, specifically young women with positive self-perceived health status (excellent/very good/good) were associated with a higher probability of not being at risk of poor mental health (AOR = 0.21 p < 0.001) controlling for the rest of the variables in the model (Table 2).

## Discussion

In this research, we convey two main findings. The *2020 Youth Survey* in Barcelona analysis pointed to a 15.3% of young women experienced period poverty at some point of her life [22]. In this research we find that these women have a higher prevalence and probability of suffering poor mental health, even after adjusting by economic and socio-demographic characteristics. This is the first study to quantify unmet menstrual product needs in a representative population sample of young women living in an urban setting and its association to wellbeing outcomes.

Most studies on the effect of period poverty have been based on low and middle-income countries’ data [52]. In those studies, period poverty has different implications since the level of poverty and deprivation are much higher, access to sanitation is more compromised and there are other contextual and cultural factors involved in menstruation [53]. In high-income countries, few studies report on the relationship between period poverty and mental health. The most relevant published research on this issue are those by Cardoso et al. [15] and Gouvernet et al. [16]. Some of their conclusions and results are consistent with our study. However, there are three substantial differences between these previous studies and the one presented here: (i) the definition of the variables used to measure period poverty; (ii) the inclusion of household income and material deprivation as control variables; and (iii) our results are based on a representative sample of the population while previous studies do not have a probabilistic-based design methodology. This is relevant insofar data collection in those two studies may also have led to a participant selection bias since they were only able to gather responses from women who had access to the internet and willingness to respond. Further, our study used a wider range of ages (15 to 34) compared to Cardoso et al. study [15], which used a rather narrower range of age (mean 20.4). Gouvernet et al. works with a wider age range (18–49) although the majority of participants (55.6%) in the online poll are young adults (18–24) [16].

Our research contributes to the literature signalling the importance of analysing some dimensions of poverty and deprivation from a gender perspective. Women in many contexts are exposed to different forms of deprivation and exclusion [54]. Our study represents an advance in identifying menstrual poverty as a distinctive dimension of deprivation, beyond the economic extent. Such evidence is key in understanding the ways in which young women manage their menstruation needs when family or personal finances are strained. It is important to highlight that the survey questions for both the material deprivation and income scale refer to the household unit, while period poverty refers to an experience of individual and gender deprivation. Our results support the idea that, under equal economic conditions and other forms of material deprivation (such as being able to afford certain expenses in terms of household’s basic needs), young women who experience difficulties in accessing and inappropriately using menstrual products are more likely to be at risk of poor mental health.

The practices used to manage, contain and clean menstrual bleeding may be conditioned by access to limited resources. An increased risk of social disclosure when money is scarce may amplify the worry, anxiety and embarrassment women and girls feel when menstruating, particularly given the shame and stigma associated with menstruation [23, 55]. In their study examining the ways in which adolescent girls in Kenya were being affected by menstrual deprivations caused by poverty, Crichton et al. [56] identified embarrassment, low mood, anxiety and fear of stigma as important consequences. Additionally, Briggs [17] identified in a qualitative study in a deprived area in UK that feelings of distress, disgust or embarrassment may lead women to self-exclusion from daily life activities such as education, sports, or work which has important implications for women’s equal opportunities.

This study is not without limitations. First, the cross-sectional design of this study makes it difficult to establish a causal relationship. Although psychological distress may result from limited access to menstrual products, the opposite relationship may also be possible, that is, women with mental health problems are more likely to experience unequal treatment, stigma, discrimination and hence poverty and period poverty. Only longitudinal studies would shed light on causality between the two variables. Second, in future studies, it would be also useful to expand the sample to cover more age groups and contexts such as rural areas that may condition access to menstrual products. Another relevant aspect that would improve future analyses is a validation of the questions we use to capture period poverty. Our measure is consistent with previous research [24], however, a validation research on this particular issue is advised. Finally, information such as the time and the frequency women are lacking access to or misuse of menstrual products have not been gathered. It is plausible that these factors may have some interaction effect on mental health.

Notwithstanding these limitations, our findings contribute to an aspect that has been little studied to date, with a higher validity when compared to previous quantitative research on the topic. This study offers new insights into tackling mental health and provides new evidence for defining and improving women’s social exclusion and mental health public policies. Social and health public services should focus on specific women’s vulnerabilities and address effective public policies such as making basic menstrual products more accessible among vulnerable populations. Some examples have emerged recently. Scotland became the first country in the world to make menstrual products free [57]; New York City provided free menstrual products in public schools, homeless shelters, and prisons [58] in 2016; the UK government introduced a scheme to enable schools and colleges in England to access funding for sanitary products [59] in 2020; in France, the government has provided free menstrual protection for college women [60]). In Spain, the Catalan Department of Equality and Feminism, together with eight other departments, has recently promoted the National Strategy for Sexual and Reproductive Rights, which includes the Menstrual Equity Plan [61]. The aim is to distribute menstrual products to high schools’ students from all over the territory in 2021. These are some recent examples of how different countries face the period poverty problem. Onwards, should be interesting to evaluate the impact of these policies on selected outcomes.

## Conclusions

This research points out that a considerable number of young women in south European urban contexts cannot access and/or misuse menstrual products for economic reasons, and this situation has a potential impact on their mental health. This research also found that under equal economic conditions and other forms of material deprivation young women who experience difficulties in accessing and inappropriately using menstrual products are more likely to suffer from poor mental health. In this regard, it is important to address at least two agendas. First, the research agenda requires rethinking poverty measurement with a gender perspective in order to identify how deprivations may overlap and aggravate the experience of poverty. Second, the policy agenda needs to take into account empirical evidence of the impact of period poverty on mental health and make steady progress in implementing effective public policies, with a gender perspective, to tackle this situation and reduce financial barriers to accessing menstrual-related products.

## Data Availability

The dataset supporting the conclusions of this article is available in the Barcelona city council repository of surveys: https://ajuntament.barcelona.cat/ca/informacio-administrativa/registre-enquestes-i-estudis-opinio.

## Abbreviations

AOR: Adjusted Odds Ratio
CI: Confidence Interval
GHQ-12: General Health Questionnaire.
